# Clinical Characteristics and Correlation of Hearing Outcomes Following Varying Courses of Repetitive Transcranial Magnetic Stimulation for Idiopathic Sudden Sensorineural Hearing Loss: A Prospective Clinical Study

**DOI:** 10.3390/jcm14207369

**Published:** 2025-10-18

**Authors:** Chao Huang, Junming Li, Ge Tan, Ling Liu

**Affiliations:** 1 Department of Geriatric Medicine and Neurology, West China School of Public Health and West China Fourth Hospital, Sichuan University, Chengdu 610041, China; huangchao2509@scu.edu.cn; 2 Department of Neurology, West China Hospital, Sichuan University, Chengdu 610041, China; tangebrain@163.com; 3 Department of Radiology, West China School of Public Health and West China Fourth Hospital, Sichuan University, Chengdu 610041, China; li18981852424@163.com

**Keywords:** idiopathic sudden sensorineural hearing loss, repetitive transcranial magnetic stimulation, treatment, tinnitus, hearing

## Abstract

**Objective**: We aimed to explore the efficacy of repetitive transcranial magnetic stimulation (rTMS) for idiopathic sudden sensorineural hearing loss (ISSNHL) and evaluate the correlation between treatment courses of rTMS and hearing outcomes. **Methods**: A prospective observational study was conducted at West China Fourth Hospital, Sichuan University, from January 2018 to January 2025. The study enrolled 339 patients (342 affected ears) diagnosed with ISSNHL. Among them, 67 patients (group A) received standard therapy combined with rTMS, while the control group (group B) received conventional therapy only. To verify the correlation between different treatment courses of rTMS and hearing outcomes, patients in Group A were divided into Group A1 (treatment courses ≤ 10) and Group A2 (treatment courses > 10). Hearing thresholds and clinical characteristics were evaluated at admission, discharge day and 6 months post-treatment. The SDRG’s criteria were used for the grading of hearing recovery. **Results**: Tinnitus (79.89% vs. 75.32%, *p* = 0.361) and sleep disorders (33.70% vs. 41.14%, *p* = 0.178) were highly prevalent among patients in group A and group B. 1 Hz rTMS significantly improved these symptoms (PSQI: 52.32% vs. 44.44%, *p* = 0.032; THI: 16.67 ± 19.41 vs. 8.22 ± 12.77, *p* = 0.002). Compared to high-tone hearing loss patients, those with low-tone loss in groupA2 showed a more rapid improvement (250 Hz: 17.66 ± 16.59 vs. 14.09 ± 15.58, *p* = 0.041; 500 Hz: 21.20 ± 18.03 vs. 17.31 ± 16.24, *p* = 0.036) than grouA1, with benefits sustained at 6-month follow-up (250 Hz: 27.79 ± 18.74 vs. 22.71 ± 18.31, *p* = 0.012; 500 Hz: 31.89 ± 19.73 vs. 26.49 ± 20.08, *p* = 0.013). **Conclusions**: rTMS at 1 Hz, administered in courses > 10 sessions, demonstrated both short-term and long-term beneficial effects in the ISSNHL. Those with low-tone hearing loss exhibit better recovery but a higher chance of relapse than high-tone loss patients. As a non-invasive approach with minimal side effects, rTMS is suitable for routine ISSNHL treatment.

## 1. Introduction

Idiopathic sudden sensorineural hearing loss (ISSNHL) is defined as a rapid-onset, unexplained, persistent sensorineural hearing loss developing within 72 h [1]. It is frequently associated with symptoms such as tinnitus, vertigo, and aural fullness, representing a common otologic emergency [2]. Reported rates of spontaneous recovery within two weeks in the absence of treatment range from 31% to 65% [2,3]. Nevertheless, 8.9% to 47% of patients experience poor audiological outcomes, including relapse following initial improvement [2,4]. Thus, there is an urgent need for a therapeutic approach with rapid onset of action and sustained therapeutic efficacy.

The pathogenesis of ISSNHL remains incompletely elucidated. Current consensus implicates multifactorial etiologies including viral infection, acute internal auditory artery occlusion, cochlear inflammation, and autoimmune mechanisms [1]. Owing to this etiological heterogeneity, treatment strategies are not standardized. Therapeutic interventions such as corticosteroids (administered systemically, locally, or in combination), vasodilators, fibrinogenolytic agents, and hyperbaric oxygen therapy demonstrate considerable variability in clinical outcomes [5]. Therefore, new strategies for treating ISSNHL are urgently needed. rTMS has emerged as a promising non-invasive neuromodulatory approach with a favorable safety profile, offering therapeutic potential for ISSNHL and other neurological disorders [6,7].

Current evidence regarding the therapeutic efficacy of rTMS for ISSNHL predominantly derives from short-term investigations, with the majority constituting cross-sectional studies of limited sample size [8,9,10]. Robust longitudinal evidence evaluating sustained treatment outcomes remains scarce. To address this knowledge gap, a prospective observational study was conducted to assess long-term therapeutic outcomes and their correlation with different rTMS treatment sessions in ISSNHL patients.

## 2. Methods

### 2.1. Research Population

A prospective observational cohort study was conducted at West China Fourth Hospital of Sichuan University from January 2018 to January 2025. Inclusion criteria comprised: (1) diagnosis of idiopathic sudden sensorineural hearing loss with consent to participate; (2) age ≥ 18 years; (3) absence of prior otologic trauma, surgery, infection, or other hearing loss-related comorbidities; (4) symptom onset-to-treatment interval ≤30 days; (5) absence of otopathology on neuroimaging. Exclusion criteria included: (1) history of neuromodulation therapies within the past year (including but not limited to rTMS, transcranial random noise stimulation (tRNS), or transcranial direct current stimulation (tDCS)); (2) comorbid neuropsychiatric disorders, intracranial tumors, implanted pacemakers, or intracranial hemorrhage; (3) retrocochlear pathology, prior otologic surgery, acoustic/barotrauma, or active/chronic otitis media; (4) patients with clinical features associated with Meniere’s disease such as two or more episodes of vertigo, fluctuating hearing loss in the affected ear, etc. Pregnant women and patients seeking clinical consultation >30 days after symptom onset were excluded. All patients were followed up at 6 months post-treatment or later.

### 2.2. Data Collection

Baseline data collection included: age, gender, comorbidities (hypertension/diabetes/hyperuricemia/hyperlipidemia), days from symptom onset to treatment, month of onset, affected ear laterality, associated symptoms (tinnitus, vertigo, aural fullness), body mass index and questionnaire results. All patients underwent pure-tone audiometry (PTA) and the tinnitus handicap inventory screening version (THI-S) [11] assessment at three standardized time points: on admission, at discharge, and 6 months post-discharge.

In accordance with the American Academy of Otolaryngology–Head and Neck Surgery (AAO-HNS) guidelines [12], hearing thresholds were expressed at four-frequency (0.5, 1, 2, and 4 kHz) by PTA. Hearing loss severity was classified using the World Health Organization standard [13]: <26 dB (within normal limits), 26–60 dB (mild-to-moderate), 61–80 dB (severe), and ≥81 dB (profound) at all frequencies.

Hearing recovery was defined as the post-treatment PTA minus the baseline PTA. Per the Sudden Deafness Research Group (SDRG) classification system [14], patients were stratified into four recovery subgroups: (1) complete recovery: hearing thresholds ≤20 dB at all frequencies (0.5, 1, 2, 4 kHz); (2) partial recovery: mean interaural hearing gain >30 dB; (3) slight recovery: the mean interaural hearing gain ranged from 10 dB to 30 dB; (4) no recovery or deterioration (<10 dB PTA improvement and PTA > 75 dB). Mean hearing threshold improvements were compared between the two groups at four test frequencies. The cure rate was defined as the proportion of patients with complete recovery, and the recovery rate as the proportion of patients with partial or complete recovery.

Tinnitus severity was assessed using the THI-S. It comprises 10 items, scored as: “Yes” = 4 points, “Sometimes” = 2 points, and “No” = 0 points. The total score ranges from 0 to 40, with higher scores indicating greater functional handicap due to tinnitus. This scale serves as a clinically validated brief screening tool for assessing tinnitus-related disability.

### 2.3. Treatment Modality

All patients received standardized therapy: Ginkgo biloba extract (35 mg/d), vitamin B_1_ (10 mg/d), and intravenous steroids (IVS) for 10 days. IVS comprised dexamethasone (10 mg/d, tapered over 10 days) to mitigate the endolymphatic hydrops, and no patients received intratympanic steroids administration. Whether to add rTMS to the treatment plan is a mutual decision made by physician and patient. According to the therapeutic protocol, patients were stratified into: Group B (standard therapy alone, *n* = 184) and Group A (standard therapy + rTMS, *n* = 158). To evaluate the dose–response relationship between rTMS session frequency and hearing outcomes, the Group A cohort was further subdivided into: Subgroup A1 (≤10 sessions, *n* = 86) and Subgroup A2 (>10 sessions, *n* = 72). rTMS targeting the temporoparietal junction (TPJ) ipsilateral to the symptomatic ear was administered for 10 consecutive days per treatment course. The stimulation was delivered using a MagPro TMS device (Magstim, Whitland, UK) equipped with a Medtronic C-B65 figure-of-eight coil (Medtronic, Minneapolis, MN, USA). rTMS was applied once daily to the TPJ. Stimulation parameters were: frequency = 1 Hz, delivering 600 pulses per session (using a pattern of 9 s on, 1 s off, over a total duration of 10 min). All patients underwent PTA assessments at three time points: on admission, at discharge, and 3 months post-discharge to evaluate hearing improvement.

### 2.4. Ethical Declaration

The study protocol was approved by the Ethics Review Committee of West China Fourth Hospital, Sichuan University (Protocol No. HXSY-EC-2020076). Written informed consent was obtained from all participants. Prior to data analysis, all medical records and patient identifiers were rigorously anonymized and de-identified in compliance with institutional review board protocols. We confirm that data collection did not commence until after the ethical approval was granted.

### 2.5. Statistical Analysis

Statistical analyses were conducted in SPSS (v24.0, IBM Corp, Armonk, NY, USA). Categorical data underwent Chi-square or Fisher’s exact testing; non-normally distributed continuous variables were analyzed via Mann–Whitney U tests. Significant predictors (univariate *p* < 0.05) were incorporated into multivariable binary logistic regression. Model outputs included adjusted ORs with 95% CIs. Two-tailed α-level was set at 0.05.

## 3. Results

A total of 365 patients diagnosed with ISSNHL were initially enrolled. After exclusion of 26 participants (5 patients with incomplete baseline data collection, 15 patients received additional treatment at outside clinics during the rTMS follow-up period, 5 patients were lost to follow-up or refused the follow-up interview, and 1 diagnosed with vestibular schwannoma during surveillance), 339 patients (342 affected ears) were ultimately included in the final analysis, among which 158 received rTMS treatment. No serious adverse events (SAEs) occurred during the treatment course.

Clinical features, including co-morbidities and demographic information, are presented in Table 1. No significant differences were observed in baseline characteristics such as age (43.44 ± 15.84 vs. 42.72 ± 15.58, *p* = 0.738), gender (53.26% vs. 51.90%, *p* = 0.829), duration of disease before visit ≤7 days (51.63% vs. 47.47%, *p* = 0.450), body mass index (BMI) (22.74 ± 1.92 vs. 23.09 ± 2.79, *p* = 0.173) between the two groups. Tinnitus (79.89% vs. 75.32%, *p* = 0.361) and sleep disorders (33.70% vs. 41.14%, *p* = 0.178) were highly prevalent among patients with SSNHL (Table 1).

We compared the hearing levels between the two groups of patients upon admission and found no significant differences in hearing level across all frequency bands (250 Hz: 56.73 ± 23.63 vs. 59.47 ± 22.83, *p* = 0.278; 500 Hz: 62.34 ± 24.52 vs. 66.20 ± 24.00, *p* = 0.143; 1 kHz: 47.64 ± 26.15 vs. 48.01 ± 25.23, *p* = 0.352; 2 kHz: 51.74 ± 29.39 vs. 51.77 ± 26.83, *p* = 0.408; 4 kHz: 76.79 ± 30.76 vs. 79.78 ± 27.12, *p* = 0.346). We found that the rTMS group exhibited significantly greater gains in low-tone hearing thresholds (250 Hz: 17.66 ± 16.59 vs. 14.09 ± 15.58, *p* = 0.041; 500 Hz: 21.20 ± 18.03 vs. 17.31 ± 16.24, *p* = 0.036) relative to the control group at discharge. Furthermore, our findings indicate that rTMS demonstrates comparable efficacy for tinnitus improvement (tinnitus on admission: 74.46% vs. 75.95%, *p* = 0.802; tinnitus at discharge: 11.41% vs. 37.12%, *p* < 0.05) (Table 2).

To evaluate the long-term efficacy of rTMS for ISSNHL, we compared the hearing thresholds and tinnitus symptoms between the two groups of patients at the 6-month post-discharge follow-up. Patients in group A demonstrated superior recovery in low-frequency hearing, while no statistically significant difference was observed between the two groups regarding high-tone hearing recovery (Table 3).

Regarding tinnitus, patients in both groups experienced recurrence after initial recovery. 5 patients recurred in Group A and 12 in Group B, showing no significant difference in recurrence rates. Further analysis revealed that among the 17 patients with recurrent tinnitus, 15 had presented with low-frequency hearing loss upon hospital admission. Furthermore, while tinnitus symptoms showed further improvement in both groups at the final follow-up, the improvement remained superior in Group A compared to Group B (7.25 ± 19.72 vs. 22.40 ± 25.36, *p* < 0.05). To evaluate short- and long-term efficacy of rTMS for tinnitus, we quantified tinnitus disability using standardized Tinnitus Handicap Inventory (THI) scores in both patient groups at three timepoints: admission, discharge, and final follow-up (Table 4)

Furthermore, to assess the relationship between rTMS efficacy and treatment sessions, patients in Group A were divided into subgroupA1 (≤10 sessions) and subgroupA2 (>10 sessions). Comparative analysis of clinical parameters, tinnitus, and hearing changes at baseline revealed no significant differences between the two patient groups across tinnitus (THI-1: 25.98 ± 16.85 vs. 25.89 ± 17.73, *p* = 0.975) and hearing level across all frequency bands (250 Hz: 42.64 ± 23.07 vs. 41.77 ± 21.57, *p* = 0.721; 500 Hz: 45.03 ± 23.85 vs. 45.17 ± 23.75, *p* = 0.998; 1 kHz: 67.01 ± 26.59 vs. 69.69 ± 26.41, *p* = 0.340; 2 kHz: 55.71 ± 31.36 vs. 54.46 ± 29.43, *p* = 0.707 4 kHz: 72.50 ± 29.51 vs. 75.03 ± 26.53, *p* = 0.493). At the follow-up endpoint, reassessment of these parameters demonstrated that patients who received 10 or more consecutive rTMS sessions exhibited significant improvements in tinnitus and low-frequency hearing, along with amelioration of sleep disturbance (Table 5).

Viral infection has been identified as one of the etiological factors for ISSNHL [15]. From an epidemiological perspective, time clustering may suggest an increased incidence of infectious causes triggering ISSNHL. To validate this hypothesis, we further investigated potential associations between SSNHL and weather conditions. Specifically, we hypothesized increased case incidence during winter-spring transition periods when viral infections or environmental etiologies demonstrate seasonal predominance, followed by declining incidence post-season. Employing meteorological season stratification [16]—spring (1 March–31 May), summer (1 June–31 August, autumn (1 September–30 November), and winter (1 December–28/29 February)—to analyze disease seasonality, we analysis of the entire cohort and found no statistically significant seasonal variation in ISSNHL incidence (spring: 52 vs. 31, summer: 39 vs. 40; autumn: 37 vs. 36; winter: 56 vs. 51; *p* = 0.306).

## 4. Discussion

Consistent with previous studies, we found that low-tone (1 Hz) rTMS can rapidly improve tinnitus and hearing in patients with ISSNHL in the short term [8]. A possible explanation is that enhanced auditory cortex activity can be observed on functional imaging in patients with tinnitus [17], while rTMS pulses of different frequencies differentially modulate cortical activity. In the motor cortex, high-frequency rTMS delivers electromagnetic pulses that transiently increase cortical excitability, whereas low-tone rTMS (i.e., 1 Hz) typically reduces neural overactivity in cortical areas [18,19,20], thereby alleviating the severity of tinnitus symptoms in patients. Furthermore, from an etiological perspective, acute internal auditory artery occlusion may be one of the causes underlying the development of ISSNHL. Studies have demonstrated that rTMS applied to the temporoparietal region can increase regional cerebral blood flow (rCBF) in the auditory thalamus ipsilateral to the stimulation, may be a possible explanation for the hearing improvement [6,21,22]. Given that low-frequency rTMS can alleviate tinnitus symptoms and hearing.

Furthermore, we observed that hearing loss at low frequencies shows a favorable hearing outcome but higher recurrence rates compared to individuals with high-frequency hearing loss. A plausible mechanistic interpretation is that low-tone hearing loss may suggest retained viability and regenerative capacity of cochlear hair cells following injury [23,24], and early use of glucocorticoids can rapidly improve inner ear labyrinth edema and low-tone hearing loss. However, these compromised hair cells demonstrate heightened susceptibility to exogenous factors such as inflammatory mediators, manifesting pronounced instability [25]. Consequently, after the corticosteroids are metabolized, follow-up observations reveal higher recurrence rates.

Previous research on rTMS has predominantly consisted of short-term studies with small sample sizes [6,8,26]. These limitations hindered the assessment of long-term prognosis and potential late complications following treatment. Consequently, rTMS as a therapeutic intervention for ISSNHL has been insufficiently emphasized in clinical recommendations. We conducted a 6-month follow-up study involving 339 patients with ISSNHL. It was found that low-tone (1 Hz) rTMS not only ameliorated hearing loss and tinnitus in ISSNHL patients in the short-term but also demonstrated sustained efficacy over the long-term. Low-frequency rTMS applied to the temporoparietal or auditory cortex may facilitate the reorganization of functional connectivity within the auditory cortex [27]. Evidence indicates that the connectivity between the auditory cortex and the limbic system, prefrontal cortex, and parietal cortex determines tinnitus severity, while connectivity between the parietal cortex and auditory cortex may influence auditory processing [28,29]. Thus, rTMS targeting these regions has the potential to ameliorate both hearing loss and tinnitus. The reorganized functional connectivity within the auditory cortex may underline the long-term therapeutic effects of rTMS.

To date, considerable heterogeneity exists in the methodologies employed across rTMS studies, particularly regarding stimulation sites and parameters. There is a paucity of empirical evidence evaluating the relative merits of these approaches. Similarly, the optimal treatment session for rTMS has received limited research attention. Our study demonstrates that the duration of rTMS stimulation represents another parameter that may influence its therapeutic outcomes. Study has demonstrated that >20 consecutive rTMS sessions are recommended for hearing improvement [6]. In our study, over 10 sessions ameliorate both hearing and tinnitus, consistent with previous investigation [30].

## 5. Limitation

Our study has several limitations. First, laterality of stimulation was not analyzed. Critical questions remain unresolved: Would ipsilateral versus symmetric cortical stimulation yield equivalent efficacy in unilateral ISSNHL? Which hemisphere requires targeting in bilateral ISSNHL? These mechanistic questions remain unexplored. Second, assessment of anxiety/depression comorbidities was conspicuously omitted. Given that limbic-auditory network integration may modulate cortical reorganization, it remains unclear whether low-frequency rTMS exerts sustained effects on hearing/tinnitus through ameliorating affective disturbances. The durability of rTMS-mediated hearing/tinnitus amelioration requires verification. Should benefits prove transient, optimal retreatment intervals and frequency-specific protocols merit exploratory trials. Crucially, multimodal neuroimaging evidence delineating cortico-network plasticity pre-/post-intervention remains lacking, which is essential to elucidate the therapeutic mechanisms of rTMS for ISSNHL on a deeper level.

Moreover, treatment responses to rTMS may vary among patients with different patterns of hearing loss. In the future, we plan to conduct a stratified analysis of the short-term and long-term therapeutic responses to rTMS in patients with specific audiometric configurations (e.g., low-frequency, mid-frequency, and high-frequency hearing loss). This will allow for more precise identification of patient subgroups that are most likely to benefit from this intervention.

## 6. Conclusions

Compared to high-tone hearing loss (HFHL) patients, patients with low-tone ISSNHL demonstrate more favorable hearing outcomes but exhibit a higher recurrence rate. rTMS at 1 Hz (>10 sessions) demonstrates efficacy in alleviating tinnitus and improving low-tone hearing thresholds in ISSNHL patients, with therapeutic effects persisting for over 6 months. As a non-invasive therapeutic modality, rTMS shows no severe treatment-related complications during long-term follow-up, supporting its integration into routine clinical management protocols for ISSNHL.

## Figures and Tables

**Table 1:** Demographic and clinical characteristics of the patients at baseline.

Factors	Standard Therapy (*n* = 184)	Standard Therapy + rTMS (*n* = 158)	*p* Value
Age (range in years, mean ± SD)	43.44 ± 15.84	42.72 ± 15.58	0.738
Gender (males, *n*%)	98 (53.26)	82 (51.90)	0.829
Course of disease			0.450
≤7 days, *n* (%)	95 (51.63)	75 (47.47)	
>7 days, *n* (%)	89 (48.37)	83 (52.53)	
Diabetes mellitus, *n* (%)	51 (27.72)	57 (36.08)	0.104
Hypertension, *n* (%)	45 (24.46)	32 (20.25)	0.367
Hyperlipidemia, *n* (%)	63 (34.24)	57 (36.08)	0.734
Hyperuricemia, *n* (%)	25 (13.59)	33 (20.89)	0.083
Ear, left (%)	96 (52.17)	77 (48.73)	0.588
Tinnitus, *n* (%)	147 (79.89)	115 (75.32)	0.361
BMI (mean ± SD)	22.74 ± 1.92	23.09 ± 2.79	0.173
PSQI, *n* (%)	62 (33.70)	65 (41.14)	0.178
Aural fullness, *n* (%)	64 (34.78)	62 (39.24)	0.423

PSQI: Pittsburgh Sleep Quality Index; BMI: Body Mass Index.

**Table 2:** Comparison of hearing improvement between group A and group B in patients upon discharge.

	Hearing Improvement (Mean ± SD)		
Frequency Band	Group A (*n* = 158)	Group B (*n* = 184)	*p* Value
	Mean Hearing Gains (dB)	Mean Hearing Gains (dB)	
250 Hz	17.66 ± 16.59	14.09 ± 15.58	0.041
500 Hz	21.20 ± 18.03	17.31 ± 16.24	0.036
1 kHz	21.68 ± 21.54	19.38 ± 18.77	0.290
2 kHz	23.29 ± 21.39	20.76 ± 21.29	0.274
4 kHz	25.38 ± 22.86	21.09 ± 22.01	0.078

Group A (standard therapy + rTMS); Group B (standard therapy alone); Hearing improvement: HLpre-HLpost; HLpre: the initial hearing level; HLpost: hearing level upon discharge.

**Table 3:** Comparison of hearing improvement between group A and group B in patients at the 6-month post-discharge follow-up.

	Hearing Improvement (Mean ± SD)		
Frequency Band	Group A (*n* = 158)	Group B (*n* = 184)	*p* Value
	Mean Hearing Gains (dB)	Mean Hearing Gains (dB)	
250 Hz	27.79 ± 18.74	22.71 ± 18.31	0.012
500 Hz	31.89 ± 19.73	26.49 ± 20.08	0.013
1 kHz	32.98 ± 23.23	30.57 ± 21.26	0.317
2 kHz	35.00 ± 22.60	32.85 ± 22.95	0.386
4 kHz	25.19 ± 22.94	21.14 ± 22.15	0.098

**Table 4:** Comparative analysis of tinnitus profiles between the two patient groups.

Factors	THI Score	(Mean ± SD)	
	Group A (*n* = 158)	Group B (*n* = 184)	*p* Value
THI-1	25.51 ± 17.48	25.03 ± 19.72	0.083
THI-2	15.61 ± 11.38	20.29 ± 19.15	0.007
THI-3	7.25 ± 19.72	22.40 ± 25.36	<0.05

THI score: Tinnitus Handicap Inventory score; THI-1: THI scores at admission; THI-2: THI scores at discharge; THI-3: THI scores at 6-month post-discharge follow-up.

**Table 5:** Comparison of demographic characteristics, auditory changes, and tinnitus outcomes between Subgroups A1 and A2 at final follow-up.

Factors	Subgroup A2 (*n* = 72)	Subgroup A1 (*n* = 86)	*p* Value
Age (range in years, mean ± SD)	48.63 ± 13.57	45.63 ± 13.41	0.166
Gender (males, *n*%)	30.23	27.78	0.861
Course of disease (day, mean ± SD)	11.16 ± 13.96	10.93 ± 11.90	0.912
Diabetes mellitus, *n* (%)	34.88	37.5	0.871
Hypertension, *n* (%)	20.93	19.44	0.638
Hyperlipidemia, *n* (%)	37.21	34.72	0.619
Hyperuricemia, *n* (%)	20.93	20.83	0.753
PSQI, *n* (%)	52.32	44.44	0.032
THI-3 score (mean ± SD)	16.67 ± 19.41	8.22 ± 12.77	0.002
Δ250 Hz (mean ± SD)	12.55 ± 13.63	21.36 ± 19.73	0.001
Δ500 Hz (mean ± SD)	15.52 ± 14.53	24.61 ± 22.65	0.003
Δ1 kHz (mean ± SD)	15.12 ± 17.61	20.72 ± 28.52	0.133
Δ2 kHz (mean ± SD)	16.40 ± 20.37	21.88 ± 24.89	0.130
Δ4 kHz (mean ± SD)	18.49 ± 21.83	24.72 ± 22.90	0.082
BMI (mean ± SD)	22.92 ± 2.58	22.81 ± 2.82	0.786

Subgroup A1: rTMS sessions ≤ 10; Subgroup A2: sessions > 10; THI-3 score: THI scores at 6-month post-discharge follow-up; THI score: Tinnitus Handicap Inventory score; PSQI: Pittsburgh Sleep Quality Index; BMI: Body Mass Index; Δ: the initial hearing level–hearing level at final follow-up.

## Data Availability

All data generated or analyzed during this study are available within this manuscript. Further enquiries can be directed to the corresponding author.

## References

[1] Schreiber B.E., Agrup C., Haskard D.O., Luxon L.M. (2010). Sudden sensorineural hearing loss. Lancet.

[2] Stachler R.J., Chandrasekhar S.S., Archer S.M., Rosenfeld R.M., Schwartz S.R., Barrs D.M., Brown S.R., Fife T.D., Ford P., Ganiats T.G. (2012). Clinical practice guideline: Sudden hearing loss. Otolaryngol. Head Neck Surg..

[3] Chandrasekhar S.S., Tsai Do B.S., Schwartz S.R., Bontempo L.J., Faucett E.A., Finestone S.A., Hollingsworth D.B., Kelley D.M., Kmucha S.T., Moonis G. (2019). Clinical Practice Guideline: Sudden Hearing Loss (Update). Otolaryngol. Head Neck Surg..

[4] Fushiki H., Junicho M., Aso S., Watanabe Y. (2009). Recurrence rate of idiopathic sudden low-tone sensorineural hearing loss without vertigo: A long-term follow-up study. Otol. Neurotol..

[5] Han X., Yin X., Du X., Sun C. (2017). Combined Intratympanic and Systemic Use of Steroids as a First-Line Treatment for Sudden Sensorineural Hearing Loss: A Meta-Analysis of Randomized, Controlled Trials. Otol. Neurotol..

[6] Zhang D., Ma Y. (2015). Repetitive transcranial magnetic stimulation improves both hearing function and tinnitus perception in sudden sensorineural hearing loss patients. Sci. Rep..

[7] Li L.P., Shiao A.S., Li C.T., Lee P.L., Cheng C.M., Chou C.C., Hsieh J.C. (2019). Steady-state auditory evoked fields reflect long-term effects of repetitive transcranial magnetic stimulation in tinnitus. Clin. Neurophysiol..

[8] Shin S.H., Byun S.W., Lee Z.Y., Lee H.Y. (2023). Short-Term Effect of Adjunctive Transcranial Random Noise Stimulation on Idiopathic Sudden Sensorineural Hearing Loss and Tinnitus: A Preliminary Study. J. Int. Adv. Otol..

[9] De Ridder D., van der Loo E., Van der Kelen K., Menovsky T., van de Heyning P., Moller A. (2007). Theta, alpha and beta burst transcranial magnetic stimulation: Brain modulation in tinnitus. Int. J. Med. Sci..

[10] Lefaucheur J.P., André-Obadia N., Antal A., Ayache S.S., Baeken C., Benninger D.H., Cantello R.M., Cincotta M., de Carvalho M., De Ridder D. (2014). Evidence-based guidelines on the therapeutic use of repetitive transcranial magnetic stimulation (rTMS). Clin. Neurophysiol..

[11] Newman C.W., Sandridge S.A., Bolek L. (2008). Development and psychometric adequacy of the screening version of the tinnitus handicap inventory. Otol. Neurotol..

[12] Committee on Hearing and Equilibrium Guidelines for the Diagnosis and Evaluation of Therapy in Menière’s Disease (1995). American Academy of Otolaryngology-Head and Neck Foundation, Inc. Otolaryngol. Head Neck Surg..

[13] Smith A.W. (1997). The World Health Organization’s Programme for the Prevention of Deafness and Hearing Impairment. Scand. Audiol. Suppl..

[14] Kanzaki J., Taiji H., Ogawa K. (1988). Evaluation of hearing recovery and efficacy of steroid treatment in sudden deafness. Acta Otolaryngol. Suppl..

[15] Wilson W.R., Veltri R.W., Laird N., Sprinkle P.M. (1983). Viral and epidemiologic studies of idiopathic sudden hearing loss. Otolaryngol. Head Neck Surg..

[16] Danielides V., Nousia C.S., Bartzokas A., Lolis C.J., Kateri M., Skevas A. (2002). Weather conditions and sudden sensorineural hearing loss. BMC Ear Nose Throat Disord..

[17] Folmer R.L. (2007). Lateralization of neural activity associated with tinnitus. Neuroradiology.

[18] Theodoroff S.M., Folmer R.L. (2013). Repetitive transcranial magnetic stimulation as a treatment for chronic tinnitus: A critical review. Otol. Neurotol..

[19] Lee H.Y., Yoo S.D., Ryu E.W., Byun J.Y., Yeo S.G., Park M.S. (2013). Short term effects of repetitive transcranial magnetic stimulation in patients with catastrophic intractable tinnitus: Preliminary report. Clin. Exp. Otorhinolaryngol..

[20] Eichhammer P., Hajak G., Kleinjung T., Landgrebe M., Langguth B. (2007). Functional imaging of chronic tinnitus: The use of positron emission tomography. Prog. Brain Res..

[21] Lefaucheur J.P., Brugières P., Guimont F., Iglesias S., Franco-Rodrigues A., Liégeois-Chauvel C., Londero A. (2012). Navigated rTMS for the treatment of tinnitus: A pilot study with assessment by fMRI and AEPs. Neurophysiol. Clin..

[22] Schraven S.P., Plontke S.K., Rahne T., Wasserka B., Plewnia C. (2013). Hearing safety of long-term treatment with theta burst stimulation. Brain Stimul..

[23] Ganesan P., Kothandaraman P.P., Swapna S., Manchaiah V. (2017). A Retrospective Study of the Clinical Characteristics and Post-treatment Hearing Outcome in Idiopathic Sudden Sensorineural Hearing Loss. Audiol. Res..

[24] Khedr E.M., Abo-Elfetoh N., Rothwell J.C., El-Atar A., Sayed E., Khalifa H. (2010). Contralateral versus ipsilateral rTMS of temporoparietal cortex for the treatment of chronic unilateral tinnitus: Comparative study. Eur. J. Neurol..

[25] Bogaz E.A., Maranhão A.S., Inoue D.P., Suzuki F.A., Penido Nde O. (2015). Variables with prognostic value in the onset of idiopathic sudden sensorineural hearing loss. Braz. J. Otorhinolaryngol..

[26] Martin A., Jana D., Lucie R., Petra H., Petra P., Manuela V., Robert J., Martin H., Zdenek S., Jiri R. (2010). Efficacy of repetitive transcranial magnetic stimulation for the treatment of refractory chronic tinnitus: A randomized, placebo controlled study. Neuro Endocrinol. Lett..

[27] Kleinjung T., Eichhammer P., Landgrebe M., Sand P., Hajak G., Steffens T., Strutz J., Langguth B. (2008). Combined temporal and prefrontal transcranial magnetic stimulation for tinnitus treatment: A pilot study. Otolaryngol. Head Neck Surg..

[28] Leaver A.M., Renier L., Chevillet M.A., Morgan S., Kim H.J., Rauschecker J.P. (2011). Dysregulation of limbic and auditory networks in tinnitus. Neuron.

[29] Hoffman R.E., Cavus I. (2002). Slow transcranial magnetic stimulation, long-term depotentiation, and brain hyperexcitability disorders. Am. J. Psychiatry.

[30] Khedr E.M., Abo-Elfetoh N. (2010). Therapeutic role of rTMS on recovery of dysphagia in patients with lateral medullary syndrome and brainstem infarction. J. Neurol. Neurosurg. Psychiatry.

